# Analysis of Urine Flow in Three Different Ureter Models

**DOI:** 10.1155/2017/5172641

**Published:** 2017-06-04

**Authors:** Kyung-Wuk Kim, Young Ho Choi, Seung Bae Lee, Yasutaka Baba, Hyoung-Ho Kim, Sang-Ho Suh

**Affiliations:** ^1^Department of Mechanical Engineering, Soongsil University, 369 Sangdo-Ro, Dongjak-gu, Seoul 156-743, Republic of Korea; ^2^Department of Radiology, Seoul National University Boramae Hospital, 425 Shindaebang-2-dong, Dongjak-gu, Seoul 156-707, Republic of Korea; ^3^Department of Urology, Seoul National University Boramae Hospital, 425 Shindaebang-2-dong, Dongjak-gu, Seoul 156-707, Republic of Korea; ^4^Department of Radiology, Hiroshima University Hospital, 1-2-3, Kasumi, Minami-ku, Hiroshima 734-8551, Japan

## Abstract

The ureter provides a way for urine to flow from the kidney to the bladder. Peristalsis in the ureter partially forces the urine flow, along with hydrostatic pressure. Ureteral diseases and a double J stent, which is commonly inserted in a ureteral stenosis or occlusion, disturb normal peristalsis. Ineffective or no peristalsis could make the contour of the ureter a tube, a funnel, or a combination of the two. In this study, we investigated urine flow in the abnormal situation. We made three different, curved tubular, funnel-shaped, and undulated ureter models that were based on human anatomy. A numerical analysis of the urine flow rate and pattern in the ureter was performed for a combination of the three different ureters, with and without a ureteral stenosis and with four different types of double J stents. The three ureters showed a difference in urine flow rate and pattern. Luminal flow rate was affected by ureter shape. The side holes of a double J stent played a different role in detour, which depended on ureter geometry.

## 1. Introduction

The ureter is part of the upper urinary system and provides a way for urine to flow from the kidney to the bladder. The shape of the whole length of the ureter could be described as a tube, a funnel, or a combination of the two. The flow of urine is partially achieved through peristalsis along with hydrostatic pressure [1]. About one to five peristaltic contractions occur per minute in the ureter, although these contractions can be disturbed by physical and mechanical irritations. Ureteral tumors and retroperitoneal fibrosis are examples of physical irritations, while a double J stent and a metallic stent inserted for ureter stones and ureteral malignancies are examples of mechanical irritations. A disturbance results in ineffective peristalsis or no peristalsis.

**Figure 1:**
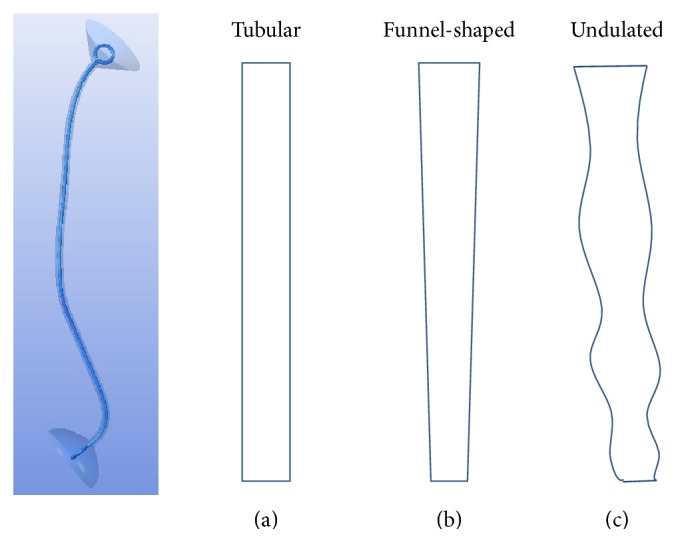
A ureter model with a double J stent. The ureter was (a) tubular, (b) funnel-shaped, or (c) undulated.

A ureteral stenosis or occlusion caused by stones or a malignancy requires a temporary or permanent urinary diversion or insertion of a ureteral stent, a thin hollow tube inserted temporarily or permanently into the ureter to prevent or treat obstruction of the urine flow from the kidney, to relieve the hydronephrosis that results from the pressure increase in the renal pelvis. The placement of a double J stent is a common treatment for stenosis, along with percutaneous nephrostomy, although various complications, such as urinary infection, migration, fracture, encrustation, and vesicoureteral reflux, have been reported with these treatments [2–4]. A double J stent is a urological catheter with two J-shaped or curled ends which are anchored in the renal pelvis and the bladder and it has multiple side holes to help urine find a detour before and after stenosed or occluded segments of the ureter (Figure 1).

Urine flow in a ureter in which there is ineffective or no peristalsis must be different from urine flow in a ureter with normal peristalsis. In this study, we investigate urine flow in the abnormal situation. We made three different, curved tubular, funnel-shaped, and undulated ureter models based on human anatomy. Numerical analysis of urine flow rate and pattern in the ureter were performed for a combination of the three different ureters, with and without a ureteral stenosis, and using four different types of double J stents, for a total of 18 CFD models.

Most CFD studies [5–9] of urine flow in the ureter have been performed with straight ureter models. However, urine in the human body flows in a curved ureter. Therefore, the studies might not reflect the real urine flow exactly; a study using a curved ureter model might produce different results.

## 2. Methods

### 2.1. Modeling of Curved Ureters with a Double J Stent

The ureter models used in this study were based on data collected from 19 men who did not have any diseases in their urinary systems. The collection of the data was described in detail in our previous studies [10, 11]. The length of the three ureter models was 226.21 mm. The diameter of the tubular ureter was 4.57 mm. Both the funnel-shaped and the undulated ureters had an upper and lower diameter of 5.69 mm and 3.59 mm, respectively. However, the undulated ureter had wall undulations along the whole ureter, which was based on the average diameters at 15 different levels along the ureteral axis [10]. According to the geometry, the volumes of the ureter lumen in the three ureters were calculated and found to be slightly different: 2.19 × 10^–5^ m^3^ in the tubular ureter, 2.22 × 10^–5^ m^3^ in the funnel-shaped ureter, and 2.21 × 10^–5^ m^3^ in the undulated ureter.

The double J stent used in the study was based on data collected from a study on a double J stent by a medical company. The stent consisted of two coils at both ends and a shaft in the middle. Each coil was round with a 10 mm diameter and had one end hole and four side holes, which were called ports to differentiate them from the side holes in the shaft. The shaft went along with the ureter and shared the same axis; the shaft had no side holes or it had multiple side holes. The inner and outer diameters of the stent were 1 mm and 2 mm, respectively. Double J stents are manufactured by various companies, and the number and geometry of the side holes in the stent depend on the manufacturer. Our previous studies [10, 11] showed that the number of side holes affected the flow rates, while the geometry of the side holes did not affect the flow rates. In this study, four different types of double J stents were made according to the number of side holes, not to the geometry of side holes. The first stent had no side holes in the shaft. The other stents had 11, 22, and 45 side holes in the shaft. The descriptions of the 18 ureter models used in the study are provided in detail in Table 1.

A stenosis in the ureter and an in-stent encrustation were made in all three ureters in the midureter, at the level of the 6th side hole of a stent with 11 side holes. A ureter model with a double J stent is shown in Figure 1. This is a combination of a tubular ureter and a double J stent without any side holes in a shaft. The different ureters are also shown in the figure.

### 2.2. Governing Equations for Fluid Flow and Numerical Simulations

The continuity equation and the Navier–Stokes equation, which were the governing equations used in the study, were converted into algebraic equations with the discretization method using the finite volume method. We analyzed the flow rates and patterns in the ureter using Ansys CFX and a pressure-based AMG coupled solver. The continuity and momentum equations are shown below:

(1) ∇•u→=0,ρ∂u→∂t+u→•∇u→=−∇p+μ∇2u→,

where *ρ*, *μ*, $\vec{u}$, and *p* are density, dynamic viscosity, velocity vector, and pressure, respectively. The Semi-Implicit Method for Pressure-Linked Equations (SIMPLE) algorithm deals with the pressure terms in the momentum equations and uses the iterative procedure in which the solution to the discretized equations is obtained [12]. The ureteral wall was supposed to be rigid and a no-slip condition was imposed on the wall [13]. When a steady solution was used, the residual of the solution of velocity and pressure was less than 10^–6^.

The urine viscosity and density applied in the study were 0.654 mPa·s and 1,003 kg/m^3^, respectively. Urine is similar to water in density and dynamic viscosity because it consists mostly of water. At a temperature of 37°C, the density and dynamic viscosity of urine are 1,003–1,035 kg/m^3^ and 0.635–0.797 mPa·s, respectively. The viscosity changes according to temperature, although the change is negligible in the range of normal body temperatures from 35°C to 40.5°C. Urine was considered a noncompressible and Newtonian fluid. Pressure was applied for the boundary condition where the inlet (97.8 Pa) and outlet (0 Pa) were specified based on the reference data [5].

Ansys ICEM was used for mesh generation. The ureter models had the same scale factor and seed size. Tetrahedron and prism meshes were used for the ports and side holes of the stents. As the number of side holes increased, the area for meshing decreased. The number of nodes and elements for a model depended on the model type; the number of nodes ranged from 1 million to 2.5 million, while the number of elements ranged from 5.7 million to 14.5 million. The grid dependency test was conducted to minimize the influence of the grid. To protect the phenomena that mesh is broken at the small side holes of a stent, global element scale factor and seed size were set as 0.975 and 0.32, respectively. Since geometry of a side hole was complex, factor of curvature based refinement was limited to 0.325. Thus, the average quality of the created grid was 0.75.

### 2.3. Evaluation Points

The flow rates and pattern in the renal pelvis, ureter, and bladder were investigated. In the stented ureter, urine flows through both the inner bore space of the stent (stent lumen) and the outer ureter space of the stent. The luminal flow rate was defined as the flow rate measured in the stent lumen; the extraluminal flow rate was defined as the flow rate in the ureter except in the stent lumen. The total flow rate was defined as the sum of the luminal and the extraluminal flow rates. The flow pattern in the stent lumen and ureter, especially around the ureteral stenosis and in-stent stenosis, was observed.

## 3. Results and Discussion

### 3.1. Total Flow Rate

**Figure 2:**
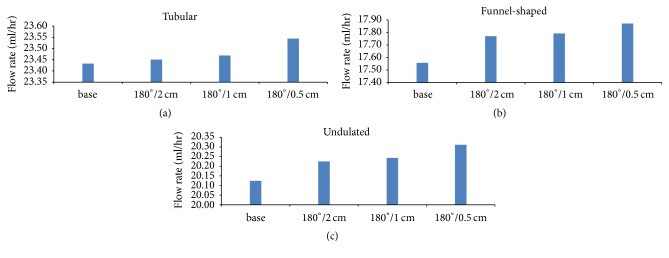
Total flow rates in three different ureters with four different types of double J stents. (a) Cases  1–4, (b) Cases  7–10, and (c) Cases  13–16.

The total flow rate in each ureter with a double J stent was 23.4~23.6 ml/h for the tubular ureter, 17.5~17.9 ml/h for the funnel-shaped ureter, and 20.1~20.4 ml/h for the undulated ureter (Figure 2). While the three ureters had the same length, curvature, and boundary condition, there was a difference in diameter and shape, which caused differences in the flow resistances and flow rates sequentially.

The total flow rate in ureters with a double J stent with side holes was higher than the total flow rate in ureters with a double J stent without side holes. Of the ureters with a double J stent with side holes, the highest rate was in the stent with 45 side holes and the lowest rate was in the stent with 11 side holes (Figure 2). These findings should be considered when choosing a ureter model for studies of ureter.

### 3.2. Individual Flow Rates

**Figure 3:**
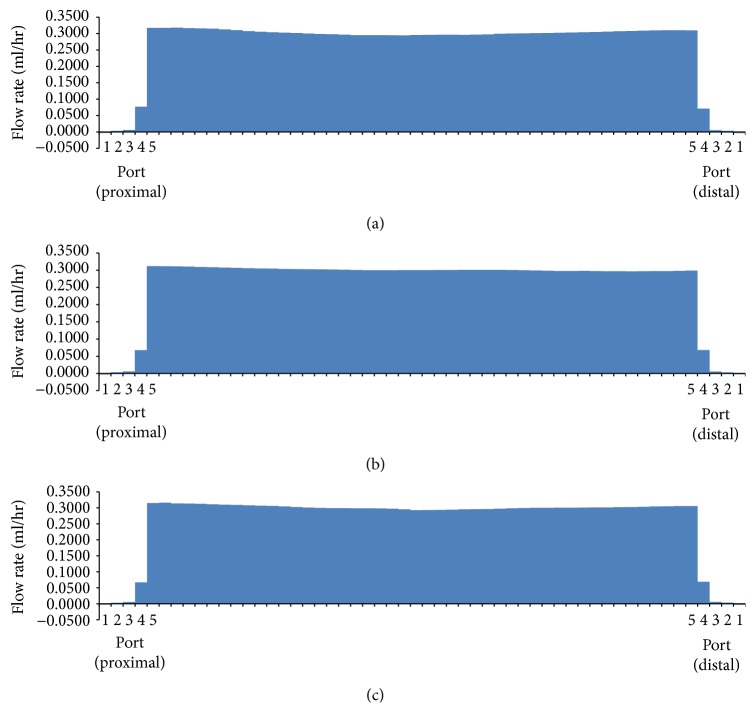
Luminal flow rates in three different ureters with a double J stent without side holes (Cases  1, 7, and 13).

The luminal flow rates in the three different ureters with a double J stent without side holes showed similar patterns (Figure 3). The urine flowed into the stent through the fourth and fifth ports of the proximal coil in the renal pelvis and maintained luminal flow; the urine then flowed out of the stent through the fifth and fourth ports of the distal coil in the bladder. Luminal flow rates were relatively constant along the whole ureter.

**Figure 4:**
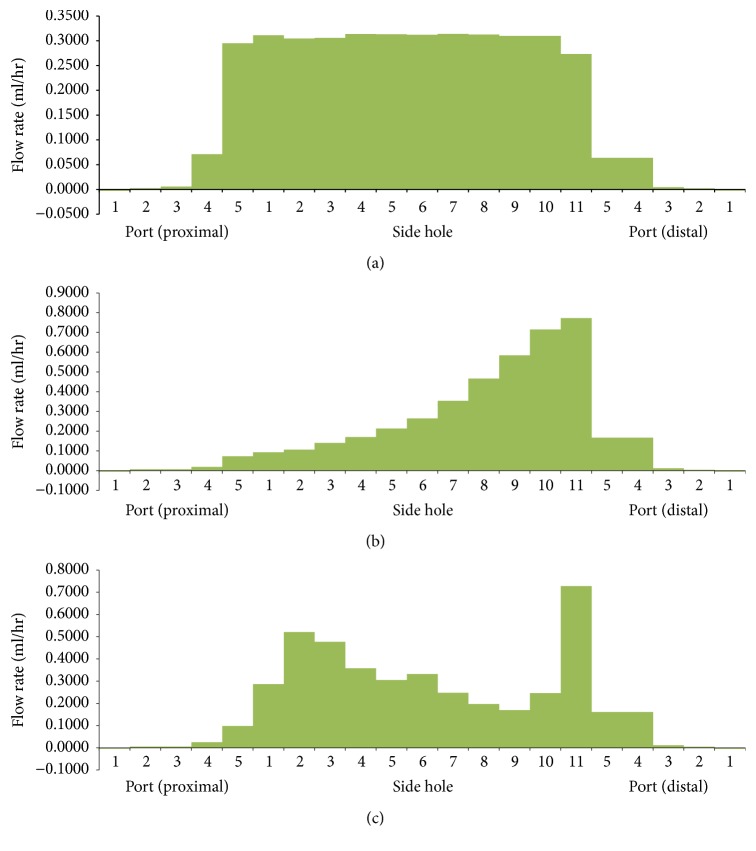
Luminal flow rates in three different ureters with a double J stent with multiple side holes (Cases  2, 8, and 14).

The luminal flow rates in the three different ureters with a double J stent with multiple side holes showed different features (Figure 4). The luminal flow rate in the tubular ureter was relatively constant along the whole ureter, which was similar to the rate in the ureter with a double J stent without side holes. However, the luminal flow rate in the funnel-shaped ureter increased gradually along the ureter and decreased sharply in the bladder; the urine flowed into the stent through the ports and side holes of the proximal coil and shaft in the renal pelvis and ureter and flowed out of the ports of the distal coil in the bladder.

The luminal flow rate in the undulated ureter fluctuated along the whole ureter. It had two peaks in the proximal and distal ureter (Figure 4). The fluctuation of the luminal flow rate could have resulted from the change in the diameter along the ureter. Diameters at the 15 levels along the ureter were 5.69, 5.27, 4.44, 3.96, 4.08, 4.25, 4.38, 4.48, 4.33, 4.42, 4.79, 4.83, 4.96, 5.11, and 3.59 mm. The two peaks and fluctuation in luminal flow rate can be seen at the two narrowest points (3.96 mm and 3.59 mm) in the proximal and far-distal ureter. Tong et al. [5] reported that there was no communication through the side holes of a double J stent in the midureter in a condition of no ureteral or in-stent stenosis; however, our study showed the existence of flow into or out of the stent through the side holes along the whole ureter. Therefore, there was communication through the side holes in the three different ureters, although minimal communication was shown in the tubular ureter. The ports and side holes of the double J stent played a role in the luminal flow at all levels of the ureter, especially in the funnel-shaped and undulated ureters.

**Figure 5:**
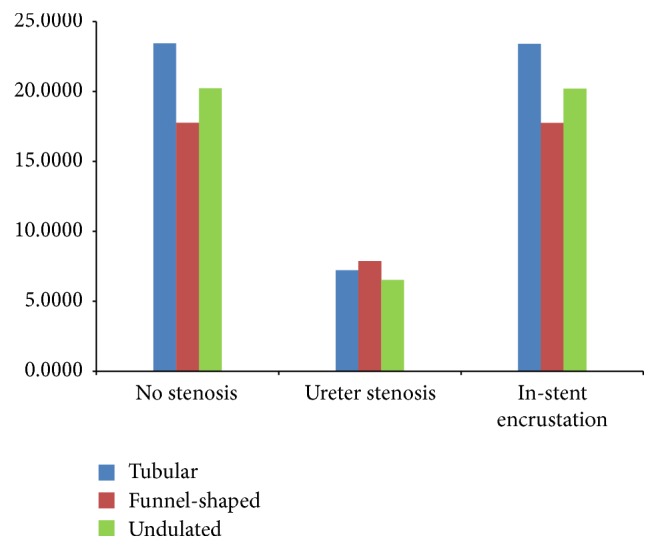
Total flow rates in three different ureters with no stenosis (Cases  2, 8, and 14), ureteral stenosis (Cases  5, 11, and 17), and in-stent stenosis (Cases  6, 12, and 18).

The total flow rates in three different ureters with ureteral and in-stent stenoses are demonstrated in Figure 5. Compared with the total flow rates in the different stented ureters with no stenosis, the total flow rates in a ureteral stenosis were smaller because of the 75% reduction in ureter inner space, which decreased the extraluminal flow rates. However, the total flow rates in an in-stent stenosis did not show any difference because the decrease in luminal flow rate was compensated by an increase in extraluminal flow rate with detours through the side holes.

The total flow rate in the tubular ureter with a ureteral stenosis was less than the total flow rate in the funnel-shaped ureter with a ureteral stenosis. The total flow rate in the undulated stented ureter with a ureteral stenosis was less than that in the tubular ureter with a ureteral stenosis. This could be explained by the diameters in the midureter where the ureteral stenosis was located. The ureter diameter in the funnel-shaped ureter was 4.64 mm (average of 5.69 mm and 3.59 mm), which was larger than the diameters in the tubular ureter (4.57 mm) and the undulated ureter (4.48 mm).

**Figure 6:**
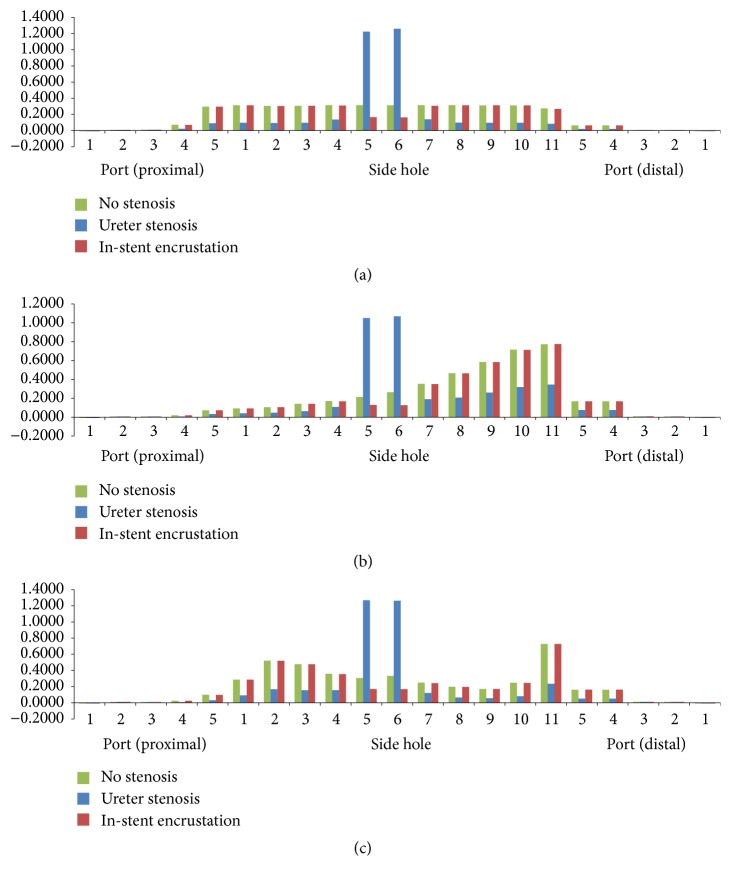
Luminal flow rates in three different ureters with no stenosis, ureteral stenosis, and in-stent stenosis. (a) Tubular ureters (Cases  2, 5, and 6); (b) funnel-shaped ureters (Cases  8, 11, and 12); and (c) undulated ureters (Cases  14, 17, and 18).

**Figure 7:**
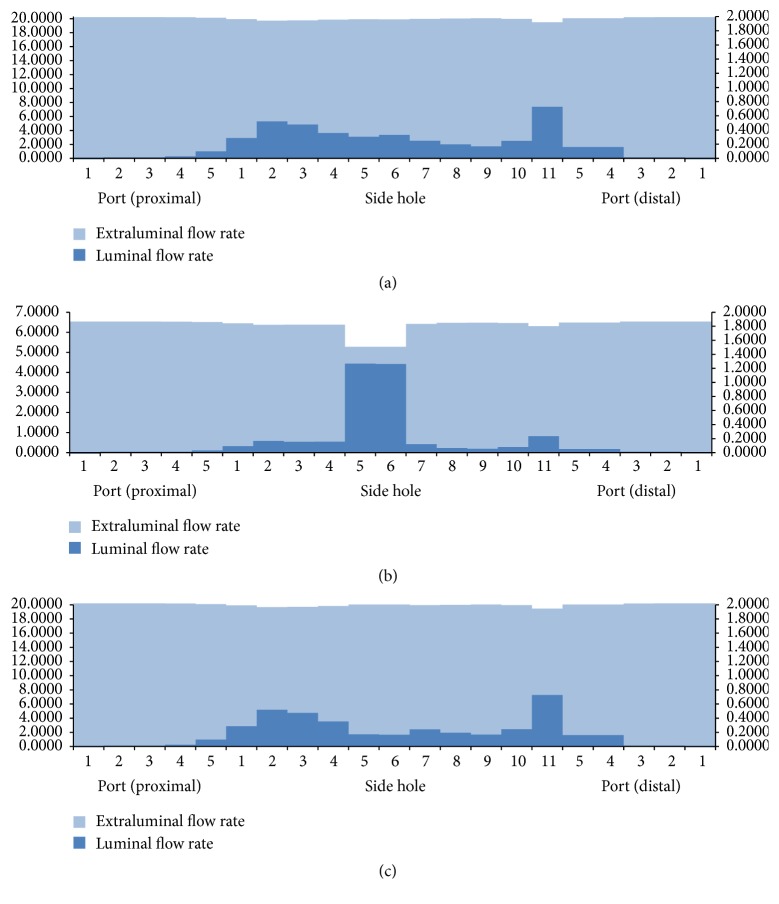
Total, luminal, and extraluminal flow rates in undulated ureters with (a) no stenosis, (b) ureteral stenosis, and (c) in-stent stenosis (Cases  14, 17, and 18).

The luminal flow rates in three different ureters with a double J stent with 11 side holes and no stenosis, ureteral stenosis, and in-stent stenosis are demonstrated in Figures 6 and 7. The pattern of flow rates in the three ureters with no stenosis was also shown in Figure 4. In the three ureters with ureteral stenosis, a peak of luminal flow rate in the midureter was superimposed on the pattern of flow rates in the three ureters with no stenosis. The peak occurred just before the ureteral stenosis and disappeared just after the stenosis. Luminal flow rates in the proximal and distal ureter were generally less than the rates in ureters with no stenosis. In the three ureters with in-stent stenosis, an abrupt decrease in luminal flow rate in the midureter was superimposed on the pattern of flow rates in the three ureters with no stenosis. The decrease occurred just before the in-stent stenosis and disappeared just after the stenosis. The luminal flow rates in the proximal and distal ureter were nearly the same as those for the ureter with no stenosis. As we mentioned in the explanation of Figure 4, in the undulated ureter, the diameter of distal ureter before the bladder was the smallest, which resulted in so much higher luminal flow rate through side hole 11 than that through the other holes except side holes 5 and 6 under ureter stenosis.

### 3.3. Flow Pattern around Side Holes

**Figure 8:**
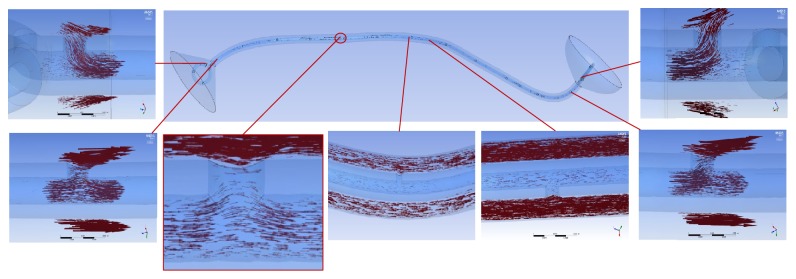
Flow vectors in proximal, mid, and distal segment of the tubular ureter with no stenosis (Case  2). Flow into and out of the stent along the ureter is not shown except small flow through the first and the last side holes.

**Figure 9:**
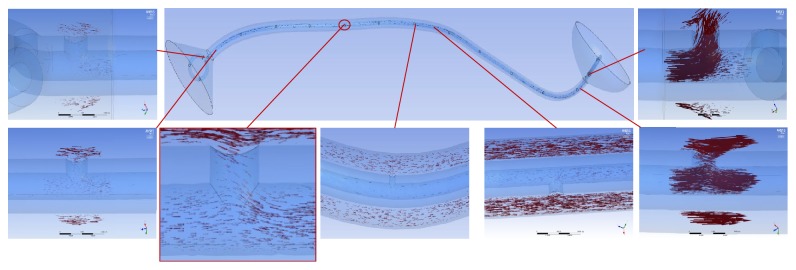
Flow vectors in proximal, mid, and distal segment of the funnel ureter with no stenosis (Case  8). Flow into the stent through all the side holes along the ureter is shown.

**Figure 10:**
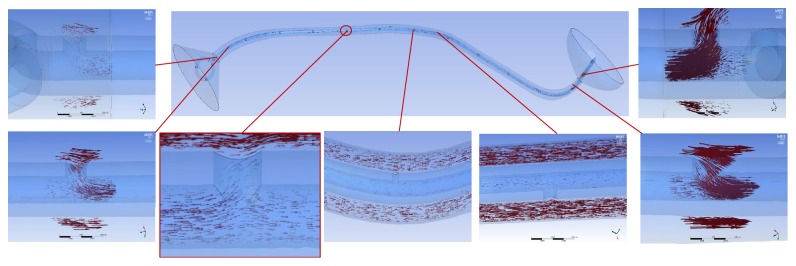
Flow vectors in proximal, mid, and distal segment of the undulated ureter with no stenosis (case  14). Flow into the stent in proximal ureter, flow out of the stent in midureter, and flow into the stent in distal ureter are shown.

**Figure 11:**
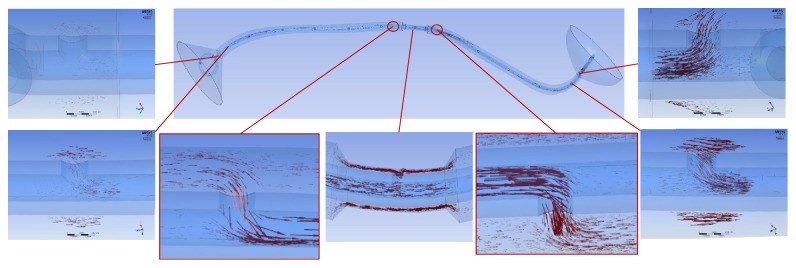
Flow vectors in proximal, mid, and distal segment of the undulated ureter with a ureteral stenosis (Case  17). Flow into and out of the stent is shown before and after the stenosis.

**Figure 12:**
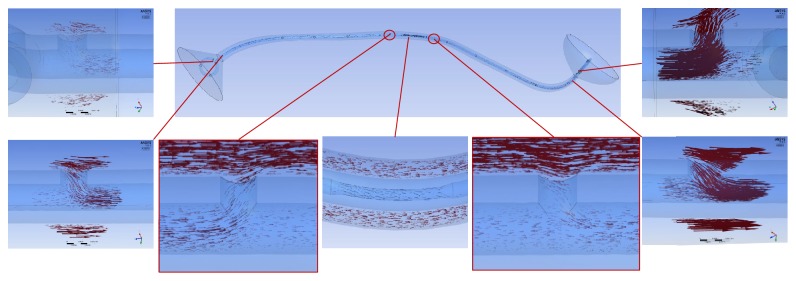
Flow vectors in proximal, mid, and distal segment of the undulated ureters with an in-stent stenosis (Case  18). Small flow out of and into the stent is shown before and after the stenosis.

In the tubular ureter, the role of the side holes in the stent shaft was limited to the first and last side holes of the double J stent; the other side holes did not play any significant role in the detour from the extraluminal to luminal spaces or the luminal to extraluminal spaces, although there was minimal fluctuation in luminal flow rates (Figure 8). However, in the funnel-shaped ureter, all the side holes played a role in the detour from the extraluminal space to the luminal space (Figure 9). In the undulated ureter, some side holes played a role in the detour from the extraluminal space to the luminal space, while others played a role in the detour from the luminal space to the extraluminal space (Figure 10). This result shows that if the ureter is not tubular, the role of the side holes in a double J stent exists regardless of ureteral or in-stent stenosis. If there is a stenosis in the ureter or in the double J stent, the role of the side holes is enhanced around the stenosis. The flow into and out of the stent was demonstrated before and after a ureteral stenosis or an in-stent stenosis (Figures 11 and 12).

Table 2 shows Reynolds numbers in the three ureter models with double J stents. When we observed flow field around side holes in the ureter models, we did not notice any significant recirculation zones affecting main urine flow. The Reynolds number is shown below:

(2) $$\begin{array}{c} Re=\frac{\rho VD}{\mu }. \end{array}$$

*ρ* is density, *V* is velocity, *D* is diameter, and *μ* is viscosity.

Table 3 demonstrates pressure at side holes along the ureter in the three ureter models. In the tubular ureter model without any stenosis, pressure decreased slowly and steadily. In the funnel ureter model without any stenosis, pressure decreased slowly and steadily in proximal and midureter and sharply in distal ureter. In the undulated ureter model without any stenosis, pressure changed sharply before and after the two narrowest points in proximal and far-distal ureter. Ureteral stenosis caused sharp pressure change before and after the stenosis in the three ureter models and in-stent stenosis caused just mild pressure change.

## 4. Conclusions

Here, CFD was capable of studying the flows through double J stents with various side holes in different types of ureter and it could be a great help in selecting the most suitable stent with an optimal position of side holes with respect to a stenosis. In this study, we found that the three different ureters showed a difference in urine flow rate and pattern. Luminal flow rate was affected by the shape of the ureter. The side holes of a double J stent played a different role in the detour depending on ureter geometry. The findings suggest that we should use a ureter model closest to the human anatomy for more accurate studies of the ureter.

## Figures and Tables

**Table 1:** Description of the ureter models used in the study.

Case	Ureter	Number of side holes	Angular position of side holes	Interval of side holes	Stenosis
1	Tubular	0	—	—	None
2	Tubular	11	0°, 180°	2 cm	None
3	Tubular	22	0°, 180°	1 cm	None
4	Tubular	45	0°, 180°	0.5 cm	None
5	Tubular	11	0°, 180°	2 cm	One in ureter (at sixth side hole), 75% stenosis
6	Tubular	11	0°, 180°	2 cm	One in stent (at sixth side hole), 30% stenosis
7	Funnel	0	—	—	None
8	Funnel	11	0°, 180°	2 cm	None
9	Funnel	22	0°, 180°	1 cm	None
10	Funnel	45	0°, 180°	0.5 cm	None
11	Funnel	11	0°, 180°	2 cm	One in ureter (at sixth side hole), 75% stenosis
12	Funnel	11	0°, 180°	2 cm	One in stent (at sixth side hole), 30% stenosis
13	Undulated	0	—	—	None
14	Undulated	11	0°, 180°	2 cm	None
15	Undulated	22	0°, 180°	1 cm	None
16	Undulated	45	0°, 180°	0.5 cm	None
17	Undulated	11	0°, 180°	2 cm	One in ureter (at sixth side hole), 75% stenosis
18	Undulated	11	0°, 180°	2 cm	One in stent (at sixth side hole), 30% stenosis

**Table 2:** Reynold number in the ureter models.

	Tubular		Funnel		Undulated	
	Maximum_Re	Minimum_Re	Maximum_Re	Minimum_Re	Maximum_Re	Minimum_Re
No stenosis	27.73	27.73	26.45	16.67	30.31	19.13
Ureteral stenosis	12.21	8.55	14.53	7.49	11.43	6.21
In-stent stenosis	27.70	27.70	26.73	16.85	30.42	19.19

**Table 3:** Pressure change at side holes in the ureter models.

	Hole 1	Hole 2	Hole 3	Hole 4	Hole 5	Hole 6	Hole 7	Hole 8	Hole 9	Hole 10	Hole 11
	Tubular [Pa]										

No stenosis	48.85	43.98	40.11	35.23	31.34	27.13	22.85	18.21	14.12	5.72	0.03
Ureteral stenosis	48.89	47.38	46.18	44.67	43.46	42.09	24.89	5.71	4.35	1.76	0.01
In-stent stenosis	48.85	43.98	40.11	35.22	31.32	27.13	18.15	14.08	5.71	1.48	0.03

	Funnel [Pa]										

No stenosis	48.88	47.78	46.51	45.07	43.15	40.84	37.95	34.29	29.33	14.63	0.06
Ureteral stenosis	48.89	48.40	47.84	47.20	46.35	45.26	30.70	15.42	13.14	6.55	0.03
In-stent stenosis	48.88	47.78	46.51	45.07	43.15	40.85	34.24	29.29	14.59	4.47	0.06

	Undulated [Pa]										

No stenosis	48.88	47.46	43.57	36.37	29.85	25.01	20.90	16.25	12.80	7.70	0.06
Ureteral stenosis	48.89	48.44	47.17	44.84	42.71	41.07	5.36	4.14	2.49	1.42	0.02
In-stent stenosis	48.88	47.47	43.58	36.40	29.89	25.07	16.23	12.80	7.71	4.39	0.06
